# Test and Prevent: Evaluation of a Pilot Program Linking Clients With Negative HIV Test Results to Pre-exposure Prophylaxis in Zimbabwe

**DOI:** 10.9745/GHSP-D-20-00444

**Published:** 2021-03-31

**Authors:** Kayla Stankevitz, Definate Nhamo, Joseph Murungu, Kathleen Ridgeway, Takudzwa Mamvuto, Rachel Lenzi, Megan Lydon, Naledi Katsande, Imelda Mahaka, Theresa Hoke

**Affiliations:** a FHI 360, Durham, NC, USA.; b Pangaea Zimbabwe AIDS Trust, Harare, Zimbabwe.

## Abstract

**Background:**

As HIV testing increases worldwide, programs are reaching individuals without HIV infection who are at risk of exposure and may be candidates for oral pre-exposure prophylaxis (PrEP). Although linkage of individuals with HIV infection to treatment is a global priority (referred to as “test and treat”), less attention is given to individuals with negative HIV test results. We developed the “Test and Prevent” pilot program to intentionally link at-risk clients with negative HIV test results to PrEP services. The intervention included risk assessment of all clients with a negative result from HIV testing (with national risk assessment tool), accompanied referral, fast-tracking, and targeting follow-up.

**Methods:**

The intervention was conducted in Bulawayo, Zimbabwe, at 6 public sector sites from October 2019 to February 2020. We collected routine monitoring data from all study sites and tracked referral completion and PrEP initiation among clients who enrolled. We conducted in-depth interviews with providers (n=12), facility managers (n=5), and female clients (n=17) to explore acceptability.

**Results:**

Among clients referred for PrEP (n=206), 98% completed their referrals and started PrEP. However, only 3% of clients who received a negative test result during the study period were referred. Low referrals stemmed from lack of screening (39% of clients with negative HIV test results were not screened) and lack of eligibility among clients who were screened (only 6% of those screened qualified as candidates for PrEP per the national screening tool). Qualitative results indicate that some providers purposefully did not complete screening with clients they felt were not at risk and that workload could have contributed to low screening uptake.

Qualitative interviews showed that Test and Prevent was acceptable among both providers and clients. Clients were happy to learn about PrEP following HIV testing, and the additional support of accompanied referrals and fast-tracking encouraged them to access PrEP and made them feel valued. Providers were burdened by workload constraints but felt that Test and Prevent was important and should be scaled to other sites.

**Conclusion:**

Intentionally linking clients with negative results to PrEP immediately following HIV testing was found to be acceptable from both provider and client perspectives, yet screening procedures need closer examination and reinforcement for the program to realize a larger impact.

## Abstract (precis)

Widespread HIV testing is identifying individuals who are not infected but are at high risk of HIV exposure. These individuals may be good candidates for pre-exposure prophylaxis (PrEP). We developed an intervention called Test and Prevent to intentionally link individuals with negative HIV test results to PrEP, which led to high rates of completed PrEP referrals and uptake.

## Teaser

**Key Findings:**

We implemented and monitored an intervention, called Test and Prevent, to intentionally link clients with a negative HIV test result to oral pre-exposure prophylaxis (PrEP).

- Most clients (98%) who were referred for PrEP immediately after their negative test result completed their referrals and started PrEP.
- The rate of screening for PrEP eligibility was low (61%). Among those screened, only 6% were deemed eligible for PrEP via the national PrEP screening tool.
- Although the subsequent referral rate (3%) was low, both clients and providers reported satisfaction with the intervention and its importance in connecting clients who test negative to PrEP.

**Key Implications:**

- Intentionally linking clients who receive a negative HIV test result to PrEP using methods from the test and treat literature could help increase PrEP uptake.
- Screening approaches for PrEP eligibility need further examination and validation to ensure they don't unintentionally screen out clients who could benefit from prevention.
- As PrEP scale-up continues, staff shortages and training needs should be addressed.

## INTRODUCTION

Provision of antiretroviral therapy (ART) as treatment for HIV has increased markedly in the past 15 years. In 2017, the World Health Organization (WHO) estimated that 59% of people living with HIV (PLHIV) globally were on ART, an increase from just 7% in 2005.^1^^,^^2^ One important strategy for initiating more PLHIV on ART is “test and treat,” which recommends that treatment start immediately following HIV diagnosis.^1^ As these approaches to HIV treatment scale up and programs prioritize intensified HIV testing, greater numbers of high-risk clients with negative test results who may be good candidates for prevention services are being identified. To date, however, little attention has been given to immediately linking clients with negative test results to prevention services, or “Test and Prevent.”^3^^,^^4^ More aggressive Test and Prevent efforts are especially warranted with an expanded range of HIV prevention options, including oral pre-exposure prophylaxis (PrEP).^5^^–^^7^ WHO recommends that oral PrEP be offered to populations at substantial risk of HIV infection, defined as having >3% HIV incidence.^5^ Since 2016, countries in sub-Saharan Africa have begun introducing oral PrEP, yet studies have shown that uptake is low among PrEP-eligible individuals.^6^^–^^8^

Evidence-based strategies that successfully link clients with positive HIV test results to treatment^9^^–^^11^ could also be effective in linking clients with negative test results to prevention, including oral PrEP. One study demonstrated that referral slips facilitate access to care among clients with HIV and reduce registration time,^12^ and many studies have shown that reminder messages sent via short message service (SMS) can be a low-cost method of promoting retention in care.^13^^–^^15^ Co-location of testing and treatment services in Zambia and Haiti have shown increases in ART initiation rates.^16^^,^^17^ Fast-tracking, a method that allows clients to bypass queues when accessing certain services, also shows promise. In Mozambique, fast-tracked clients with a positive result from HIV testing were more than twice as likely to start ART within 1 week than those offered standard care.^18^

These approaches linking clients from HIV testing to follow-up services hold potential for Test and Prevent interventions, but there are complexities unique to prevention services that require attention. While clients receiving an HIV-positive result may be motivated to act immediately,^19^^,^^20^ individuals with an HIV-negative result may feel less urgency to seek prevention services. Some research has even shown an association between HIV-negative test results and subsequent increased risky behavior.^4^^,^^21^^,^^22^ Further, no single solution exists for clients with HIV-negative results, and the appropriate prevention option may change over time.^23^ Adaptation is required and more evidence is needed on the feasibility and acceptability of Test and Prevent programs.

The Ministry of Health and Child Care (MOHCC) in Zimbabwe is among the HIV program leaders seeking evidence about linking clients with HIV-negative test results to prevention. The goal of the current evaluation was to assess the feasibility of a Test and Prevent program to intentionally link such clients to oral PrEP. Given the lack of evidence around Test and Prevent, we aimed to assess the percentage of clients referred for PrEP who completed the referral as well as to examine qualitatively the acceptability and feasibility of Test and Prevent strategies from both clients' and providers' perspectives.

The goal of the current evaluation was to assess the feasibility of a Test and Prevent program to intentionally link clients without HIV to oral PrEP.

## INTERVENTION

We designed the Test and Prevent pilot intervention in consultation with the MOHCC and United States Agency for International Development mission in Zimbabwe. We aimed to identify a package of highly effective linking interventions that could be affordably implemented at scale within the national program, assuming the package proved to work effectively. We aligned the intervention with national guidelines for HIV testing and PrEP delivery.^24^ Components of the intervention are summarized in Table 1.

**TABLE 1.** Components of the Test and Prevent Pilot Intervention in Zimbabwe

Intervention Component	Current National Guidelines for PrEP^24^	Test and Prevent Pilot Intervention
Risk assessment	Clients with negative HIV test results who are at substantial risk of HIV should be screened for PrEP eligibility using the RAST (see Box)	Use the RAST to screen all clients with negative HIV test results immediately after delivering results
Counseling and referral	Clients with negative HIV test results should be proactively linked to prevention services	All clients who are determined to be at risk based on the RAST receive Detailed counseling about oral PrEP using the national PrEP fact sheet Referral to oral PrEP services, including referral slip A prevention services card, detailing the prevention services available at the facility
Accompanied referrals with fast tracking	Not part of national guidelines for PrEP	Women referred for PrEP are given the option of having a provider immediately accompany them to PrEP services at the time of referral. When accompanying a client, the provider facilitates fast tracking, allowing the client to skip the queue.
Follow-up	Not part of national guidelines for PrEP	Clients not completing their PrEP referral in 2 weeks receive reminder messages via phone call, WhatsApp, or SMS (based on client preference at enrollment).

Abbreviation: PrEP, pre-exposure prophylaxis; RAST, Risk Assessment Screening Tool; SMS, short message service.

Briefly, providers were asked to complete a risk assessment using the national Risk Assessment Screening Tool (RAST) with all clients who had negative HIV test results. Additional information about the RAST and other risk assessment tools used in Zimbabwe are included in the Box. Clients deemed potential candidates for PrEP based on the RAST were provided with more information about the study, and those who were interested completed informed consent and were enrolled. Clients who were not considered at risk or chose not to enroll could still receive counseling and prevention services. However, they were not offered accompanied referrals, fast-tracking, or follow-up messages, and no data were collected about them to inform study outcomes. After enrollment, participants were counseled about PrEP and given referrals. After referral, women were offered accompanied referrals and fast-tracking. Finally, clients who did not complete referrals were given reminders via phone or messaging.

BOX **HIV Risk Assessment Tools in Use in Zimbabwe**

National guidelines in Zimbabwe promote 2 risk screening tools, the Risk Assessment Screening Tool (Figure 2) and the Adult Screening Tool (Figure 3). Neither tool was developed for the pilot intervention. Rather, they are standard components of HIV services in Zimbabwe, but provide important context to understand the HIV testing population and pre-exposure prophylaxis (PrEP) referrals.

Before testing, per national guidelines, eligibility for HIV testing should be assessed using the Adult Screening Tool. This tool uses a series of questions to determine whether a client is eligible for HIV testing. A client is considered eligible if they meet any of the following criteria:

- Reports experiencing poor health in the past 3 months
- Considers their own risk of HIV to be mild, moderate, or severe
- Has experienced symptoms of a sexually transmitted infection
- Has a partner or parent living with HIV

In addition to the Adult Screening Tool, national guidelines also promote the use of the RAST after an HIV-negative test result to determine whether a client should be offered PrEP or postexposure prophylaxis or be considered at risk for acute HIV infection. The RAST also assesses HIV risk but uses different criteria. Based on the RAST, a client is a candidate for PrEP if they meet any of the following criteria:

- Has had vaginal or anal sex with 2 or more people in the past 6 months
- Has not used a condom every time they had sex in the past 6 months
- Has had a sexually transmitted infection in the past 6 months
- Has a partner living with HIV

While the study enrolled both men and women, we recognized that additional barriers exist for women accessing prevention services. Given budget and workload constraints, we provided the more time-consuming accompanied referrals and fast-tracking intervention only to women. We also chose to only conduct in-depth interviews with female clients because they received the most intensive intervention and could therefore provide insights on Test and Prevent.

Additional barriers exist for women accessing prevention services, so the more time-consuming accompanied referrals and fast-tracking intervention were provided only to women.

## METHODS

### Setting

Zimbabwe began PrEP delivery in 2015, initially offering PrEP in nongovernmental organization clinics, then expanding to government-supported sites. Unlike some countries that targeted PrEP rollout only to specific populations, PrEP was available in Zimbabwe to all individuals without HIV who were at substantial risk of exposure.

This study was conducted in Bulawayo, Zimbabwe. As the second largest city in Zimbabwe, Bulawayo is an urban center with HIV prevalence estimated at 17.9%, which is higher than the national average of 14.6%.^25^

The intervention was implemented in 6 public sector sites in Bulawayo: 5 clinics and 2 HIV testing points within a central hospital (the antenatal care [ANC] clinic and the outpatient department). Sites were purposely selected based on availability of PrEP services and client volume for HIV testing. Although we originally intended that the intervention be delivered approximately 2 months after PrEP was first introduced to these facilities, delays in the PrEP supply chain resulted in PrEP and the intervention being rolled out simultaneously. The pilot intervention was implemented and evaluated at these sites from October 2019 to February 2020. Due to concerns around the availability of medicines and sustainability, PrEP demand creation activities in Bulawayo did not take place until October 2020, well after the study period.

The location of HIV testing and PrEP services varied across study sites. Most sites, with the exception of the ANC service delivery point, deliver PrEP services in the opportunistic infections (OI) unit, which is in a separate part of the health facility compound from HIV testing. As such, clients referred to PrEP from HIV testing needed to walk to the OI unit to learn more about PrEP and receive their drugs. Alternatively, at the ANC service delivery point, PrEP services were integrated into the ANC department, and clients received HIV testing and PrEP at the same location. Clients at this site did not require accompanied referrals.

### Outcome Measures

The primary outcome measure was the percentage of clients referred for PrEP who completed that referral. The secondary outcome was the percentage of clients given a PrEP referral who initiated PrEP. We also qualitatively explored intervention feasibility and the facilitators and barriers clients experienced regarding PrEP access and provision.

Unlike some countries that targeted PrEP rollout only to specific populations, in Zimbabwe, PrEP was available to all individuals without HIV who were at substantial risk of exposure.

### Data Collection and Data Analysis

We used a prospective cross-sectional design, comprising collection of both qualitative and quantitative data. To assess the primary and secondary outcomes, we asked providers to complete study forms, introduced through a 1-day training, to track client referrals and completion of referrals. We limited the number and length of these study forms to simulate real-world service delivery as much as possible, and we therefore collected limited information about participants, including age, gender, name, contact information, whether they received a referral, whether they completed the referral, and whether they accepted the offer of PrEP (initiated PrEP). We also collected routine monitoring data on the number of HIV tests performed and number of positive HIV tests, to serve as the denominator for measuring PrEP uptake. The study team provided supportive supervision for completion of study forms to improve data quality.

We conducted in-depth interviews with clients and providers to assess their perceptions of the intervention. Providers were selected purposively to include 1 facility-in-charge per facility as well as providers who were most involved in administering the intervention. Since we were particularly interested in the experiences of women accessing PrEP services, qualitative interviews with clients were only with female clients who received a PrEP referral. Women that participated in the intervention were selected for qualitative interviews using stratified random sampling by site and referral completion status. We attempted to interview 3–5 women from each site who were given a PrEP referral and completed that referral, and another 3–5 women from each site who were given a referral but did not complete the referral.

Trained qualitative interviewers conducted in-depth interviews following a structured guide. Interviews covered topics including feasibility and acceptability of intervention components, impact on provider workload (for providers), overall impressions of intervention implementation (for facility managers), and recommendations for future improvements. Interviews also included close-ended questions about time allocated to each intervention component (for providers), and prior knowledge of PrEP and experiences with HIV prevention and intervention components (for clients). Interviews were held in private locations (generally in study facilities) and were conducted in Shona, Ndebele, or English (the 3 most common languages in Zimbabwe) depending on the preference of the respondent. Interviews lasted on average 34 minutes with providers, 19 minutes with facility managers, and 12 minutes with clients. Interviews were audio recorded, transcribed, translated to English (if necessary), and reviewed for accuracy.

Transcripts were uploaded into NVivo v.12 for coding and analysis.^26^ Research analysts coded the data using a codebook developed according to the interview guide. To assess intercoder agreement, analysts coded 11% of transcripts independently and then compared results, discussed, and updated the codebook to reflect agreed-upon coding approaches. After coding, code reports were analyzed into memos around key study themes.

### Ethics

Ethical approval for the study was obtained from the Protection of Human Subjects Committee at FHI 360 (IRBnet number 1447486) and the Medical Research Council of Zimbabwe in Zimbabwe (approval number MRCZ/A/2488). All providers and study staff who collected study data were trained in research ethics. Participants provided written consent to participate in the study.

## RESULTS

### PrEP Screening, Referrals, and Uptake

**FIGURE 1:**
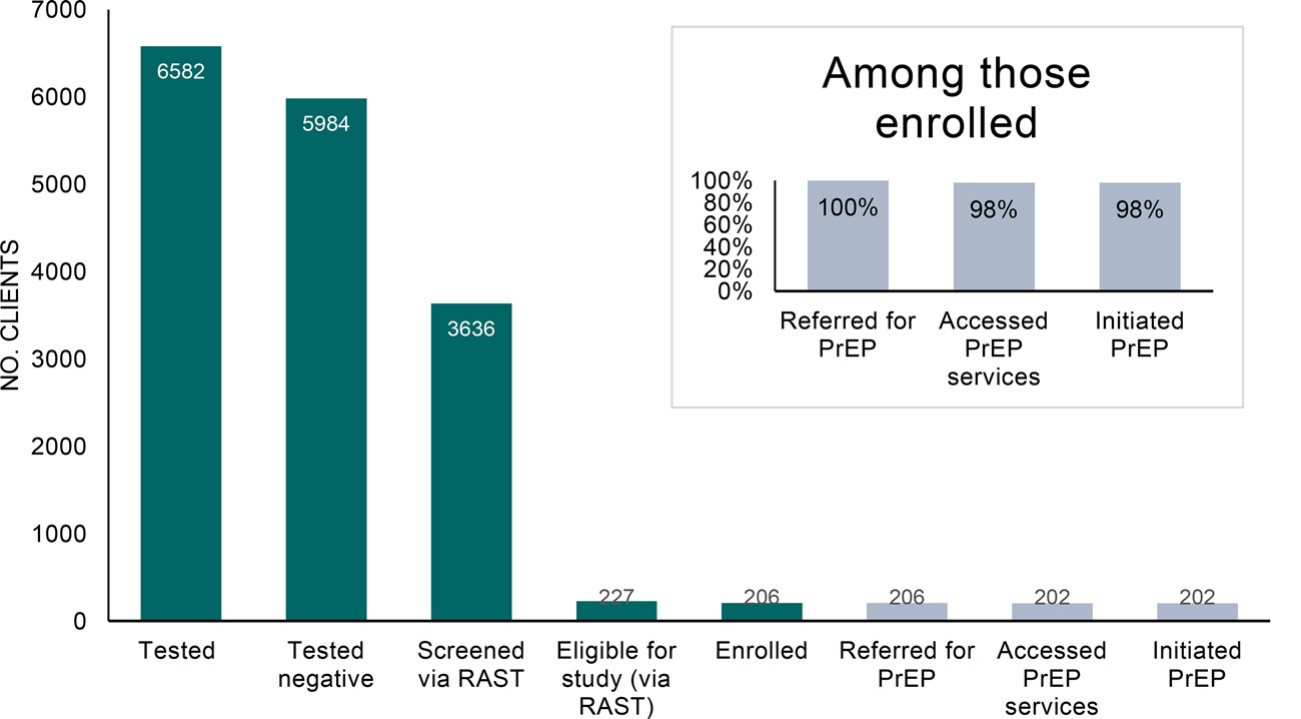
Number of Clients at Each Stage of the HIV Test and Prevent Intervention, Zimbabwe Abbreviations: PrEP, pre-exposure prophylaxis; RAST, Risk Assessment Screening Tool.

Data were collected from November 4, 2019, to February 29, 2020. A total of 6,582 individuals underwent HIV testing at the study sites during the 17-week study period (Figure 1). Of these, 91% received negative results. Only 61% of the individuals with negative results were screened for HIV risk using the RAST, and of these, only 6% were deemed at risk of HIV, and thus invited to enroll in the study.

**FIGURE 2:**
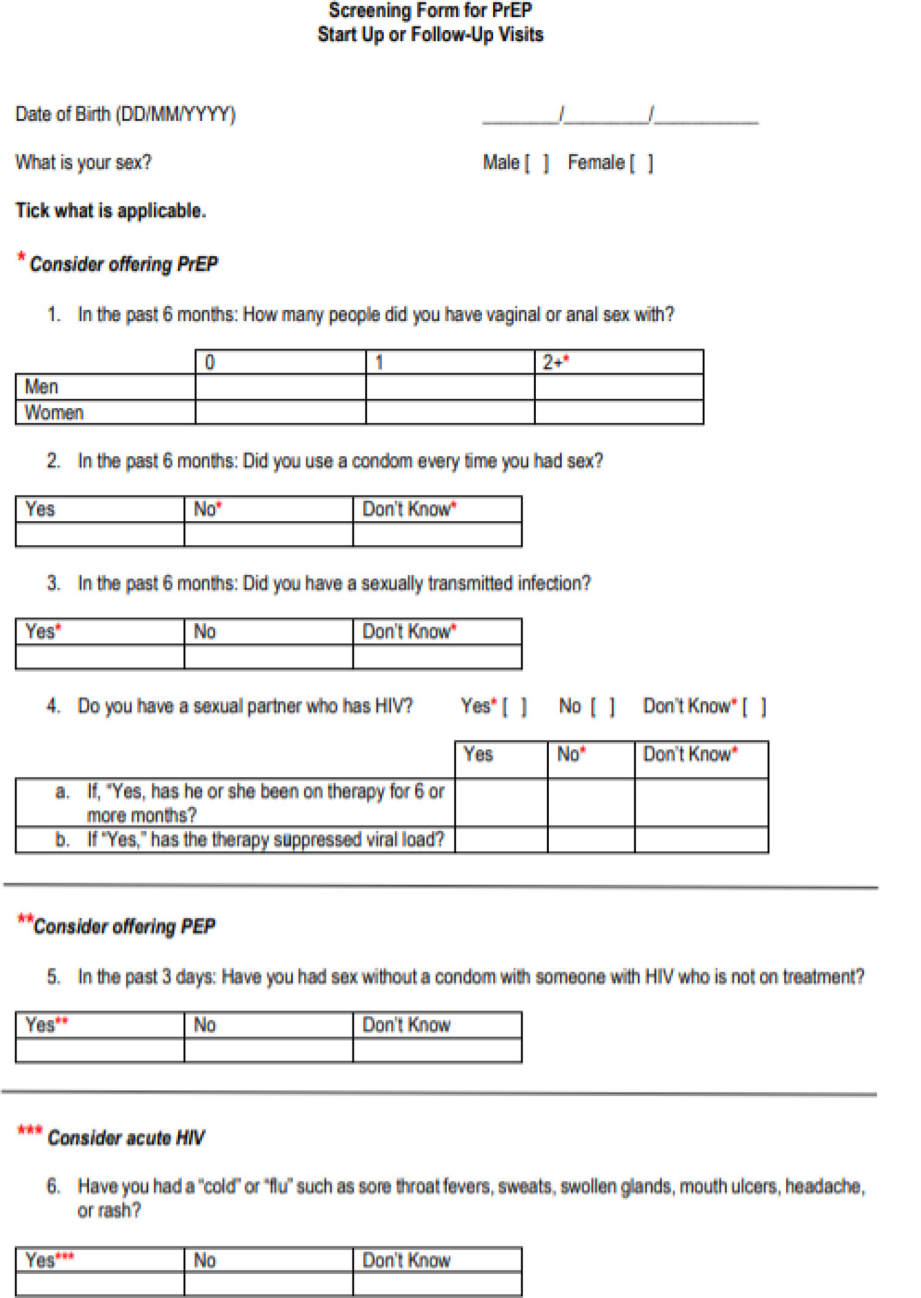
Risk Assessment Screening Tool Used in Zimbabwe for HIV Risk Screening Abbreviations: PEP, postexposure prophylaxis; PrEP, pre-exposure prophylaxis.

**FIGURE 3:**
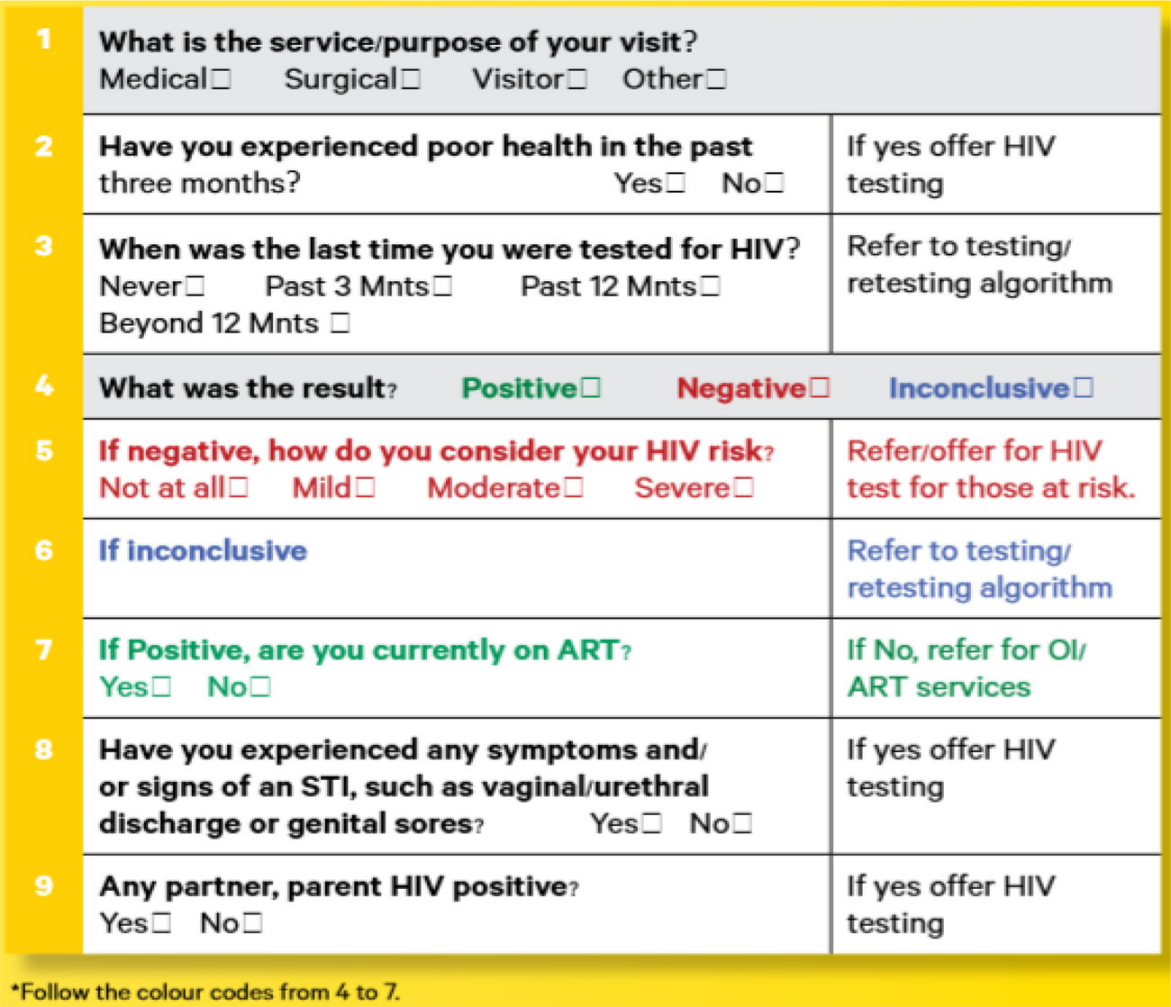
Adult Screening Tool Used in Zimbabwe for HIV Risk Screening Abbreviations: ART, antiretroviral therapy; OI, opportunistic infections unit; STI, sexually transmitted infection.

Of those deemed eligible via the RAST, 91%, or 206 individuals, agreed to participate in the study and were enrolled. Reasons for declining enrollment included being interested in PrEP, but not the study; wanting more time to think about the study; being too busy; and wanting to discuss enrollment first with their husband or partner.

Among the 206 clients who enrolled, 77% identified as cisgender women, 23% cisgender men, and <1% transgender women (Table 2). The mean age was 32 (SD: ± 11) years old. Of those enrolled, 50 were adolescent girls and young women (i.e., female and between the ages of 15–24). Most clients were from sites delivering PrEP at the OI unit (n=167); 39 clients were from the ANC service delivery point that offered PrEP onsite.

**TABLE 2.** Age and Sex of Enrolled Participants in Test and Prevent Pilot Intervention in Zimbabwe (n=206)

Characteristics	No. (%)
Age, years	
15–18	13 (6.3)
19–24	46 (22.3)
25–29	49 (23.8)
30–39	50 (24.3)
40–49	31 (15.0)
≥50	17 (8.3)
Sex	
Female	158 (76.7)
Male	47 (22.8)
Transgender	1 (0.5)

After enrollment, all participants were told about PrEP immediately at the site of HIV testing and referred to PrEP services. Of the 206 participants who received referrals, most (n=202) accessed PrEP services, and all participants who accessed PrEP services accepted the offer of PrEP. Among the 4 individuals who did not complete the referral, 2 were from sites that offered PrEP at the OI unit, and 2 were from the ANC service delivery point. They were all women aged 21–30.

Of the 206 participants who received referrals, 202 accessed PrEP services, and all participants who accessed PrEP services accepted the offer of PrEP.

### Qualitative Sample Characteristics

Qualitative interviews were conducted with 12 providers, 5 facility mangers, and 17 female clients. Providers were mostly women (n=11), and the sample was composed of registered nurses (n=7), primary counselors (n=4), and 1 clinic referral facilitator. Facility managers were all women. Clients had a mean age of 35 years old (range 20–64). All had received PrEP counseling, accepted a referral, and completed their referrals. While we tried to interview the 4 female clients who did not complete their referrals, all declined.

The results below are structured by clinical step involved in the PrEP process and incorporate both qualitative and quantitative findings to provide a more holistic understanding of the intervention.

### Risk Assessment Screening Tool

Although monitoring data indicate that a third of the clients with negative HIV test results were not screened using the RAST, just over half of the providers interviewed described completing the RAST with every client with a negative result. Others noted that they used the RAST with a subset of clients: those who asked for PrEP or those presumed to be at risk because they are in a serodiscordant relationship or identify as a member of a key population. One provider explained:

*Those patients who are saying the partner is positive and [they are] negative … that is where I would want to use the risk assessment tool*. —Registered nurse, acting Sister-in-Charge

In interviews, many providers expressed concerns about the sensitivity of RAST questions. They felt that clients were uncomfortable answering questions about their sexual activity—especially the first question of the tool, which asks about anal sex—and were not always forthcoming. Providers described utilizing counseling skills and their own judgment, instead of following the RAST questionnaire directly. As explained by one provider:

*When you are conducting the risk assessment, some of the clients will be shy to tell you. Especially the first question: “How many people did you have vaginal sex with?” So, you try to put it in a way that they can understand so they are free to share. “Sister, here you can tick 2 plus.” Especially for elderly people … I just create a good rapport with the client before doing anything. Just try to be friendly. Then if it's an elderly person, I humble myself. If it's a young person in their mid-twenties, I bring myself to their level. Then I try to ask.* —Registered nurse and midwife

Some providers were concerned that the RAST may miss some clients who are at risk, including clients with a partner who is not monogamous. Others described needing to probe to identify risk not covered in the tool.

Despite these challenges, every provider and facility manager interviewed said they would recommend that other facilities use the RAST, with one provider qualifying that counselors needed to use their own “assessment” not just the questionnaire. Most described the RAST as a helpful guide to administer and document a risk assessment, and some expanded that it would be more difficult to determine who is appropriate for PrEP without this kind of guidance.

*I think out of all the things, [the RAST] will make life easy when administering PrEP because after you have done the screening you know everything about the patient, such that when you are now prescribing the tablets, the client won't ask other questions that will lead you back to deciding whether I should give PrEP or not.* —Registered nurse

Despite challenges, every provider and facility manager interviewed said they would recommend that other facilities use the RAST.

### Use of the RAST and the Adult Screening Tool

Results showed a disconnect between results of the Adult Screening Tool and the RAST. Presumably, all clients who received HIV testing were determined to be at risk based on the Adult Screening Tool prior to testing. In qualitative interviews with facility managers we confirmed that all sites were using the Adult Screening Tool, with the exception of the ANC site where HIV testing is offered to all pregnant women. Yet among HIV testing clients who were later screened with the RAST, only 6% (227 clients) were deemed at risk, suggesting that 94% of these clients were considered at risk from the Adult Screening Tool, but not at risk based on the RAST.

#### PrEP Counseling and Referral

Providers reported that counseling about PrEP often happened in conjunction with conducting risk assessment via the RAST. When asked how they determine which clients to counsel on PrEP, most providers noted that they primarily counseled serodiscordant couples or clients in serodiscordant relationships, although some mentioned that they provided PrEP counseling to all clients who have HIV-negative test results and/or perceived themselves to be at risk of HIV. Tracking results indicated that not all clients with negative test results were being informed about PrEP, however, nor was the RAST used systematically to determine which clients to inform about PrEP.

Providers described that while counseling clients after HIV testing and receipt of negative results, they informed clients about PrEP, discussed clients' HIV risk, and addressed fears and concerns. Some mentioned that clients typically had low pre-existing knowledge or awareness of PrEP, and the counseling process was consequently more challenging or time intensive than standard counseling about HIV risk reduction. Others noted that clients may have concerns about PrEP, particularly due to the fact that it was a novel product in Bulawayo, or that clients may be reticent to share information about HIV risk behaviors during PrEP counseling. Echoing provider accounts, several interviewed clients stated that they learned about PrEP for the first time through the counseling following HIV testing.

Echoing provider accounts, several interviewed clients stated that they learned about PrEP for the first time through the counseling following HIV testing.

Most providers reported they were successful in addressing clients' questions and concerns and in improving clients' knowledge about PrEP, noting that clients “didn't know anything” about PrEP before but “are now knowledgeable.” One provider described the importance of tailoring PrEP counseling to clients' specific situations, noting that no single strategy may work well across all clients:

*… you should treat each client differently because as clients come they have different presentations, they have different scenarios, so what works well is what the clients presents you with and should act the way the client presents to you. If it's a sex worker, you counsel according to their sex work. If they are in a serodiscordant relationship you counsel according to that … So, you can't really say this [single strategy] works well in counseling.* —Registered nurse

All providers responded favorably to the idea of recommending that other facilities implement PrEP counseling after HIV testing and described that it should be “a priority,” noting that it is an “important” and “beneficial” service that could “empower” and inform clients. Likewise, all clients interviewed said receiving PrEP counseling and referral immediately following their test results was acceptable, and many spoke of it being a relief to find out PrEP was an option.

*I felt very happy knowing that I am going to get help, so that I do not get this disease …. Knowing my husband's [HIV] status, I was just happy knowing that at least one of us can be safe and look after the kids in case anything happens to him.* —Client

#### Accompanied PrEP Referrals and Fast-Tracking

Providers who were responsible for accompanied referrals indicated that they offered accompanied referrals to female clients that they referred; however, some mentioned that they did not do this consistently with all female clients as prescribed. Likewise, providers stated that fast-tracking of PrEP clients did not occur universally across facilities or clients but did take place in most cases.

Some providers shared apprehensions about accompanied referrals, including concerns about leaving other clients waiting and spending a long time guiding clients from one location to the next. At the same time, providers noted that collaboration between providers, especially of different cadres, and short distances between referral locations eased these challenges. Likewise, providers had concerns about the interruption of service delivery when having to prioritize a PrEP client, and the lack of fairness to other patients who may be waiting for equally important services. Clients who did not receive fast-tracking noted that the wait time and queue were relatively short.

Some providers indicated that being accompanied to the location of PrEP services at the OI unit may incite stigma and deter clients from completing referral, explaining that:

*You see someone frowning saying, I have to go to OI now, if they see me there they will assume I'm infected*.

Despite some providers' concerns, no clients mentioned this issue.

Most clients who received accompanied referral recalled having a positive experience. They appreciated having help to “know where to go” and assistance with communication. Some noted that accompanied referral made them “happy,” and one participant explained that,

*If you have someone with you it helps you to confidently go through the whole process without turning away.* —Client

Likewise, clients remarked that fast-tracking made them feel valued. One participant admitted experiencing negative treatment from other patients whom she was allowed to bypass, but she nevertheless appreciated the service.

Providers highlighted that accompanied referrals and fast-tracking help ensure that PrEP clients are prioritized and gives clients the “support and encouragement” they need. Both providers and clients overwhelmingly agreed that accompanied referrals decreased loss to follow-up. One provider explained that it “helps you not lose the clients in the process.” Many clients who received accompanied referral directly stated that they would not have gone to the PrEP services to complete their referral without this assistance. They identified numerous barriers that accompanied referral helped them overcome, including fear of the medication, anxiety expressing oneself to providers, lack of understanding the counselor, not knowing where to go, long queues, and negative partner attitudes. Even clients who felt they would have completed the referral on their own expressed that “the accompaniment made it much better,” highlighting the support this service can provide across clients.

Providers highlighted that accompanied referrals and fast-tracking help ensure that PrEP clients are prioritized and gives them needed “support and encouragement.”

At the same time, clients who were initiated in the same room where HIV testing was conducted (at the ANC clinic where PrEP was integrated into HIV testing) considered this the ideal scenario, explaining that,

*You do fall out along the way. Then you go to the other place, that side there's a queue and you end up meeting people you don't want to see. So it's best you put the two of them in the same room and the process is just done.* —Client

#### Workload

We asked providers how the intervention's additional tasks affected their workload. Just over half of providers reported “a little” impact on workload, while the remaining providers reported a large increase in workload. We also asked providers to report the amount of time it took to complete each study component. Quantitative responses are summarized in Table 3.

**TABLE 3.** Reported Time to Complete Different Components of Test and Prevent Intervention, Zimbabwe

Study Component	Average Time (Range), Min
RAST	10 (3–30)
Counseling and referral following HIV testing	17 (5–30)
Accompanied referral and fast tracking	13 (5–45)

Abbreviation: RAST, Risk Assessment Screening Tool.

Providers reported the RAST taking an average of 10 minutes to complete. Some felt extra time required for the RAST was minimal, as counseling and risk assessment were already taking place, stating, “It's just an added prevention tool to the HIV prevention program, which is already existing.” Other providers felt it did increase the time spent with each client significantly. One provider described:

*Conducting risk assessment? It's like I'm a PC [Primary Counselor]. I've got a queue of people waiting to be tested …. So, by the time I finish with that client conducting the risk assessment, they will start sharing, and you can't stop the patient. They will start sharing their story and at the same time answering the risk assessment tool. Then I tell them about PrEP … but my queue is stuck.* —Primary Counselor

Providers reported counseling and PrEP referral taking an average of 17 minutes. Most providers felt counseling is time consuming because clients are not well informed about PrEP and had questions.

*… I think it [PrEP counseling] needed a little bit of extra time than the normal counseling. 'Cause it depended on the client, 'cause some clients had fears so you really had to go out of your way to explain and address those fears … [but] when you start it was actually flowing and we were able to do everything.* —Registered nurse

A few providers felt counseling did not require additional time, because they were already counseling clients on other prevention options, stating, “Counseling is counseling.” A provider at one site that implemented group PrEP education sessions in the waiting room described that such sessions helped reduce the time needed for individual counseling.

Time to complete accompanied referrals varied. Most sites were clinics and therefore did not have a large distance between HIV testing and the OI unit where PrEP was delivered. This contributed to shorter times: all sites except one reported times of 4–5 minutes. One provider explained:

*Being a clinic set up, well the services wouldn't be really far apart so it wouldn't really take much of your time to move from one room to give the patient to the next service provider.* —Registered nurse

The provider reporting that accompanied referrals require more time explained that the process involves more than simply walking to the site of services. Fast-tracking can take additional time if a provider is not ready to receive the client. This participant said:

*So, it's about 45 [minutes] because it's going there and coming back. There you are not just going to say here it is, you make sure that you have handed the patient to the right person.* —Registered nurse

Most providers mentioned workload challenges, often reiterating this concern. When asked if there were times when they were unable to complete the risk assessment, some providers described feeling rushed because of workload constraints, stating, “some clients were not screened.” Others mentioned not probing as much as they would have liked, explaining that,

*The queue will be very long, so sometimes, like the risk assessment, you have to shorten it depending on the first answers you had during the pretest counseling …. For some it will be a disadvantage, because you won't be able to probe enough. Somebody would have given you shallow answers. You wouldn't have time to go deeper because of the queue outside and you will be doing everything by yourself*. —Primary counselor

When asked if they were sometimes unable to complete the risk assessment, some providers described feeling rushed because of workload constraints.

Similarly, when discussing counseling challenges, providers said that their facilities are “overwhelmed” and are dealing with a “shortage of staff.” When asked about how to improve PrEP counseling following HIV testing, providers had few suggestions about modifying the content or process of PrEP counseling, but rather suggested that additional staff be allocated to the task. Likewise, when providers were asked about challenges related to referrals and fast-tracking, most providers responded that they were short-staffed. Providers highlighted that having insufficient staff at any point in the PrEP referral chain—from counselors, to providers who do accompanied referrals, to nurses initiating PrEP—causes a backlog of clients, increases wait times and client frustration, and hinders the facility's ability to carry out the full PrEP referral process.

Compounding the challenge of general workforce shortages, only a few providers per site were trained on the study procedures, and few were trained to deliver PrEP services. Consequently, no providers were available to replace trained providers who were on leave, went out to lunch, or were not scheduled to work. In interviews, providers described these constraints as directly affecting patient care, even resulting in some PrEP clients leaving without being seen.

### PrEP Acceptability and Participant Recommendations

Clients were overwhelmingly in support of PrEP delivery, describing it as “a good thing” and “a good idea.” Clients described feeling “happy” and having “a peaceful feeling inside my heart” in response to learning about or using PrEP. Others described feelings of reassurance, saying, “my life is safe” and that “[PrEP will] help me stay negative.”

Despite general acceptance, both clients and providers discussed recommendations to improve PrEP delivery. The most common recommendation among both clients and providers was the need for community sensitization. Clients felt there was little knowledge about PrEP in the community, and outreach could help reach more people as well as make disclosure easier for PrEP users. Providers felt that community sensitization was important to legitimize PrEP, ensure clients have heard of PrEP, and increase reach.

Despite general acceptance, both clients and providers noted the need for community sensitization about PrEP.

Providers shared multiple recommendations around workload. In addition to requesting more staff, providers felt hiring one person who could be responsible for all stages of PrEP delivery could help streamline Test and Prevent, reduce waiting times and stigma, and ensure all interested clients receive PrEP services. Along those lines, providers and some clients suggested that integrating PrEP services within the same room as HIV testing could eliminate time moving between these services, reduce patient volume at the OI unit, and decrease the stigma of clients with negative HIV test results accessing services with PLHIV.

## DISCUSSION

Effective, scalable interventions are needed to link at-risk individuals with negative HIV test results to PrEP. As rates of HIV testing have grown, such individuals are increasingly being identified. Some countries, including Zimbabwe, already have guidelines for screening clients immediately following HIV testing and providing referrals for PrEP, yet the rates of referral and completion of referrals are not well documented. Building on the “test and treat” literature, we designed and evaluated an intervention to proactively link clients with negative HIV test results to the site of PrEP services, immediately after they receive their result.

This study identified high rates of completed PrEP referrals (98%) and PrEP uptake (98%) among clients with negative results referred to PrEP after testing. These findings suggest that when a referral was made, linkage to PrEP services using the Test and Prevent approach piloted in this study was highly successful. However, we found that very few clients were offered PrEP overall; as such, these outcomes could reflect selection bias in PrEP referrals. While Test and Prevent is a rather new concept, a similar intervention to intentionally link HIV testing clients to PrEP was recently conducted as part of the SEARCH study, among a much larger population.^7^ They found that only 27% of those referred initiated within 90 days. More evidence is needed to confirm that the referral rates and outcomes documented in our small-scale study in Zimbabwe can be sustained at scale, particularly when higher volumes of clients are offered PrEP referrals. Further, while high PrEP uptake is promising, PrEP effectiveness is contingent upon adherence and continuation. This study did not measure adherence or continuation and therefore we cannot determine whether high uptake led to continued use or sustained HIV prevention among study participants.

Providers and some clients suggested that integrating PrEP services within the same room as HIV testing could save time moving between these services, reduce patient volume, and decrease client stigma.

In contrast to the high rates of linkage to PrEP that we observed, our results indicate a substantial gap in screening and referrals. Only 3.4% of clients with negative HIV test results were referred to PrEP during the study period. Per Zimbabwe's national guidelines, the RAST should be used to determine if a client is appropriate to refer for PrEP. However, more than one-third of clients with negative HIV tests were not screened using the RAST during the study. Interviews suggest that some providers purposefully avoided screening clients who they felt were not at risk, and that some providers did not screen when their workload was too heavy. Perceived barriers such as clients' potential discomfort with the content of the RAST could have biased selection of clients for screening and counseling. In the context of national workforce shortages, gaps in implementation are not surprising.^27^

More surprising, of those clients screened with the RAST, only 6% were considered at risk for HIV and appropriate for PrEP referral. Most of the study sites (with the exception of the ANC site) used the Adult Screening Tool to screen clients prior to HIV testing. Our findings demonstrate a disconnect between the results of these 2 tools. Presumably, most clients who received HIV testing were determined to be at risk based on the Adult Screening Tool, yet among those who were later screened with the RAST, only 6% were deemed at risk. This finding has major implications for PrEP provision because it means most clients with negative HIV test results may not be identified as eligible for PrEP referral per Zimbabwe guidelines.

Approaches for screening clients prior to offering PrEP require further study. Judicious provision of PrEP services requires accurate risk identification; accordingly, a variety of assessment tools have been developed to attempt to identify individuals at greatest risk.^28^^–^^30^ However, the content of these tools varies widely. In 2018, a review of existing risk assessment tools found that among 24 tools currently used to assess risk, only 3 were validated.^31^ As such, it is difficult to understand whether unvalidated tools appropriately quantify and predict risk. While quantitative tools are appealing, some oral PrEP stakeholders have suggested that risk tools should not define strict cutoffs for eligibility, but rather should serve as a process to initiate a conversation around the potential benefits of oral PrEP.^31^ This sentiment was echoed by providers in this study who felt that adhering too strictly to the questions defined in the RAST could lead to a lack of identification of at-risk clients. The RAST requires further examination, as well as validation, to improve its use as a tool for channeling clients from HIV testing to PrEP services.

We also identified provider concerns that clients were not comfortable discussing sensitive risk assessment questions that are part of the RAST. Other studies have shown that some clients have challenges communicating with providers about sexual behaviors,^32^ and that conversely, providers too can be uncomfortable and will avoid taking sexual histories.^33^^,^^34^ Further refinement of the RAST to address the sensitivity of some questions, as well as encouraging providers to administer it in a more conversational manner, could help address this. However, given the low rate of utilization of the RAST, provider training to address discomfort and bias in discussing risk behaviors may be necessary. The number of times clients have to discuss risk could also be reduced. Clients accessing PrEP through study sites needed to discuss risk before testing while completing the Adult Screening Tool, after HIV testing while completing the RAST, and after completing the referral before being given PrEP. Centralizing PrEP services could be explored to reduce the burden on clients of having to discuss risk multiple times.

Despite challenges with the overall number of referrals, our qualitative findings demonstrate high acceptability of the pilot Test and Prevent program among clients who were referred. While much of the positive response in interviews can be attributed to the availability of PrEP in general, and not necessarily the linkage intervention, most clients did express support for accompanied referrals and fast-tracking. This complements other studies that have demonstrated acceptability of accompanied or “escorted” referrals among clients accessing HIV treatment or family planning services.^12^^,^^35^ Little evidence is available on the use of “fast-tracking” in clinic settings, and while clients in this evaluation found it acceptable, some providers expressed concerns that it would unfairly result in other clients having longer wait times. Further assessment of the equity of fast-tracking may be needed before considering offering the service at scale.

Despite challenges with the overall number of referrals, the pilot Test and Prevent program was deemed highly acceptable by clients who were referred.

Despite the acceptability of Test and Prevent among providers, those interviewed did raise concerns with workload, often citing pre-existing staff shortages. Of note, Zimbabwe's persistent national shortage of health care workers was particularly acute during the study period.^36^ Stemming from the economic crisis that devalued their salaries, health care workers in the country had come to an agreement with the MOHCC to only work a limited number of days a week until their salaries increased. As a result, during the study, many staff were working reduced hours or a reduced number of days. Nonetheless, our results show that additional cascading of the national PrEP training would be needed to ensure that adequate numbers of staff were trained at each site to provide coverage. These human resource challenges directly affected PrEP delivery, resulting in some clients not being screened and others leaving without accessing PrEP. To prevent staff burnout and ensure quality services for clients, staff shortages and training needs should be addressed before further scale-up.

Both providers and clients felt that further community sensitization of PrEP was necessary. Targeted community sensitization can improve PrEP awareness and reduce misinformation.^37^ Community sensitization should not target just potential PrEP users, but also partners and family members to foster support.^38^^,^^39^ While community sensitization has the potential to make it easier for providers to counsel on PrEP, higher PrEP demand has workload implications that need to be considered in this context.

Community sensitization should not target just potential PrEP users, but also partners and family members to foster support.

### Limitations

We recognize important limitations of this work. Most significantly, the lack of an experimental design prevents us from making conclusions about the effectiveness of Test and Prevent because we do not know how many individuals would have completed their PrEP referrals without the added support. We did not track PrEP continuation and therefore cannot determine whether PrEP initiations using the Test and Prevent approach led to long-term PrEP use or HIV prevention. As sustained PrEP use is ultimately needed for PrEP to be effective, continuation is an important metric to assess in future Test and Prevent studies. Results suggest that some providers only screened individuals for PrEP eligibility if they perceived them to be at high risk, and this selection bias limits the generalizability of these findings by not necessarily capturing perspectives of all HIV test clients who could benefit from PrEP. This intervention was implemented when PrEP was first being introduced in these facilities and community sensitization in Bulawayo was minimal. We acknowledge that provider and client perspectives about the Test and Prevent intervention might be different if PrEP was a well-established prevention service. Many participants first learned about PrEP during the study, making it difficult to determine whether opinions expressed in interviews reflect positive attitudes towards the intervention, or positive opinions about PrEP in general. We interviewed only female PrEP clients and therefore can only comment on the acceptability of the intervention among women. Further, all 4 individuals who did not complete their PrEP referrals declined interviews; as a result, qualitative results are limited to the experiences of those who completed their PrEP referrals and may be favorably biased. The intervention took place at a small number of sites that were already experiencing workforce shortages, which makes it difficult to conclude which challenges were a result of the intervention versus a result of existing constraints on providers' time.

Despite these limitations, this study highlights the potential of the Test and Prevent program to link at-risk clients who have received negative HIV test results to PrEP and to ultimately lead to PrEP uptake. It also identifies important considerations from the perspectives of clients and providers for PrEP scale-up as a whole. The study is timely. Given that many countries are expanding PrEP delivery to the general population, this work highlights important considerations for stakeholders at multiple levels of PrEP planning.

## CONCLUSION

Intentionally linking clients who have received negative HIV test results to PrEP immediately following testing was found to be acceptable from both provider and client perspectives. Results show that the intervention was successful in encouraging clients who were given a PrEP referral to access PrEP services (98%) and those that completed the referral all initiated PrEP. Yet the screening procedures to identify good candidates for PrEP use need closer examination. Questions in the national screening tool should be reviewed for sensitivity, and repetitiveness between PrEP screening and risk screening prior to HIV testing should be addressed. Training should reinforce that screening for PrEP needs to be conducted consistently with all clients, and clients should not be excluded due to providers' perceptions of their risk. Improved screening for HIV risk will be essential for Test and Prevent to realize a larger impact on PrEP uptake in this setting.

## Notes

**Peer Reviewed**

**First published online:** February 26, 2021.
